# Lipopolysaccharide Upregulates Neuroinflammation, Oxidative Stress Responses, and Peroxiredoxins in Depression Models

**DOI:** 10.1002/brb3.71231

**Published:** 2026-01-28

**Authors:** Zhifang Zhang, Nanshi Li, Mingkun Liang, Fangyan Qin, Qijing Qin, Qing He, Kaihua Wang, Xueli Shi, Ying Jiang, Hui Qin

**Affiliations:** ^1^ Department of Neurology Guangxi International Zhuang Medicine Hospital Affiliated to Guangxi University of Chinese Medicine Nanning Guangxi China; ^2^ Mental Health Department The First Affiliated Hospital of Guangxi Medical University Nanning Guangxi China; ^3^ Department of Neurology Ruikang Hospital Affiliated to Guangxi University of Chinese Medicine Nanning Guangxi China; ^4^ Guangxi University of Chinese Medicine Nanning Guangxi China

**Keywords:** depression, lipopolysaccharide, neuroinflammation, oxidative stress responses, peroxiredoxins

## Abstract

**Introduction:**

Depression is a chronic psychiatric disorder and belongs to one of the leading causes of suicide worldwide. Peroxiredoxins (Prdxs) play a critical role in scavenging excess reactive oxygen species (ROS) and mitigating oxidative stress. However, the role and underlying mechanisms of Prdxs in depression have not been fully illustrated.

**Methods:**

We carried out lipopolysaccharide (LPS)‐induced ICR depression mice and BV2 cell inflammation models. Seven days after LPS‐induction, behaviors in ICR mice were assessed by open field test (OFT), sucrose preference test (SPT), and forced swim test (FST), and inflammatory factors levels in serum were quantified via ELISA. The expression levels of Prdxs were evaluated using immunohistochemistry (IHC), western blotting (WB), and RT‐qPCR. In LPS‐induced BV2 cells, inflammatory factor levels in the supernatant were measured by ELISA. Nitric oxide (NO) levels were detected by biochemical assay. ROS levels were detected via fluorescence signal intensity. Prdxs expression levels were analyzed using WB and RT‐qPCR.

**Results:**

In LPS‐induced ICR mice serum and BV2 cells supernatant, interleukin‐1 beta (IL‐1β), tumor necrosis factor‐alpha (TNF‐α), and transforming growth factor‐beta1 (TGF‐β1) levels exhibited significant elevation (*p* < 0.05). In the hippocampus region of LPS‐induced mice and LPS‐induced BV2 cells, significant upregulation of Prdx1, Prdx2, Prdx4, and Prdx5 levels was observed (*p *< 0.05). The ROS and NO levels in LPS‐induced BV2 cells also significantly increased (*p *< 0.05).

**Conclusions:**

This study revealed that Prdx1, Prdx2, Prdx4, and Prdx5 were elevated in depression models, which might relate to the occurrence of neuroinflammation, coupled with upregulation of oxidative stress responses. This study provided new strategies for the treatment of depression.

## Graphical abstract

Peroxiredoxins exhibited upregulated expression during the progression of lipopolysaccharide‐induced depression companied with neuroinflammatory and oxidative stress responses, suggesting peroxiredoxins might serve as novel strategies for the treatment of depression.

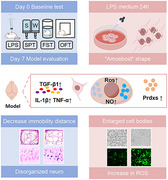

## 1 Introduction

Depression is characterized by a range of symptoms, including anhedonia, appetite disturbances, weight fluctuations, cognitive dysfunction, and even severe suicidal ideation (O'Rourke et al.2023; Ramsey et al. 2021). According to the epidemiological surveillance from the World Health Organization (WHO), about 3.8% of the population experiences depression, including 5% of adults (4% among men and 6% among women) and 5.7% of adults over 60 years old. Major depressive disorder (MDD) affects approximately 350 million individuals worldwide, leading to about 800,000 suicides annually, which constitutes 14.3% of all suicide‐related mortality (Cao et al. 2021; Mao et al. 2024; Shen et al. 2024).

The pathogenesis of depression involves multifactorial mechanisms, including encompassing monoamine neurotransmitter dysregulation, hypothalamic pituitary adrenal (HPA) axis dysfunction, neuroinflammation mediated by cytokines (particularly tumor necrosis factor‐alpha [TNF‐α] signaling), abnormalities in the nitric oxide (NO)/cyclic guanosine monophosphate (GMP) pathway, mitochondrial autophagy–apoptosis interactions, gene–environment interactions that modulate neural plasticity, etc. (K. Zhang et al. 2023; Drevets et al. 2022; T. Z. Wang et al. 2023; Lu et al. 2023). Neuroinflammation is closely associated with psychiatric disorders such as depression, and inflammatory factors play a pivotal role in the development of emotional disorders (Wohleb 2016). However, the specific underlying mechanisms still need further exploration.

Microglia, the resident immune cells of the central nervous system (CNS), play a critical role in neuroinflammation and participate in brain defense against adverse stimuli, neuronal activity, and synaptic plasticity (Wohleb 2016). Lipopolysaccharide (LPS) can activate microglia, leading to pro‐inflammatory responses and depressive‐like behaviors (Y. Li et al. 2024). Reactive oxygen species (ROS) are essential for cell signaling pathways and defense against microorganisms invasion. However, excessive ROS and depletion of antioxidant defense disrupt redox homeostasis to activate pro‐inflammatory signaling cascades, which could lead to cellular necrosis (Bhatt et al. 2020). In depression, the imbalance between ROS overproduction and insufficient antioxidant capacity contributes to oxidative stress, leading to brain dysfunction and abnormal neuronal signaling processes (Sies and Jones 2020; Y. Wang et al. 2018).

Peroxiredoxins (Prdxs), a highly conserved family, play a crucial role in clearing excessive ROS and reducing oxidative stress (T. Qiu et al. 2025; Zhou et al. 2024). In patients with irritable bowel syndrome (IBS), increased anxiety and depression have been verified to be associated with elevated serum Prdx1 and TNF‐α levels (Y. Zhang et al. 2021). Prdx2 could reduce the production of ROS and participate in regulating various signaling pathways in neurons by catalyzing hydrogen peroxide (H_2_O_2_), thereby protecting neurons against oxidative stress and inflammatory injury (J. Liu et al. 2020). However, spillage of Prdx2 could accelerate brain damage after stroke by activating inflammatory responses (J. Liu et al. 2020). Prdx4 has been reported to alleviate the microvascular inflammation and the infiltration of destructive neutrophils and pro‐inflammatory macrophages into brain tissue after cerebral ischemia/reperfusion (I/R) (Yang et al. 2021). Prdx5 could regulate Ca^2+^‐neurophosphatase‐dependent dynamin‐related protein 1 (Drp1) dephosphorylation by clearing cytoplasmic ROS, thereby regulating LPS‐induced mitochondrial division (Park et al. 2016).

So far, the relationship and mechanisms between Prdxs and depression remain unclear. This study aimed to investigate the role of Prdx1, Prdx2, Prdx4, and Prdx5 in neuroinflammation and depression based on the LPS‐induced ICR mouse depression model and the BV2 cell inflammation model to provide potential new strategies for depression treatment.

## 2 Material and Methods

### 2.1 Establishment of LPS‐Induced Depression Model in ICR Mice

Twelve adult male ICR mice (7–8 weeks old, weighing 18–22 g) were obtained from Hunan Silek Jingda Experimental Animal Co.,Ltd. (Hunan, China). The animals were housed under controlled conditions with 12 h light/dark cycle, 65% humidity, a temperature of 22 ± 2°C, and free access to purified water and standard chow.

Mice were randomly divided into two groups (*n* = 6, per group): the control group (Saline, Yuyuan, Pubei, China) and the LPS group (LPS solution, 1 mg/kg/day, SIGMA, Saint Louis, USA). All mice received consecutive intraperitoneal injections once every day, lasting for 7 days, 0.1 mL/day.

### 2.2 Behavioral Testing of Open Field Test

Behavioral tests were conducted 24 h after the final intraperitoneal injection. To eliminate the animal sickness factors and avoid the biases due to sickness and blunted behaviors induced by LPS, the open field test (OFT) was performed according to the previously developed protocol (Zhao et al. 2019). Specifically, mice were adapted to the experimental room for 1 h and then were individually placed in the OFT measuring chamber of 45 cm × 45 cm × 30 cm. A total of 5‐min video was recorded to monitor mice locomotor activity. The total distance covered by mice was measured and analyzed.

### 2.3 Behavioral Testing of Sucrose Preference Test

Each mouse was individually housed in a separate cage equipped with two drinking bottles and standard chow for a 2‐day acclimatization period. One drinking bottle contained purified water, and the other with 2% sucrose solution. To prevent positional bias, the positions of bottles were alternated every 12 h. The formal test began at 8:00 a.m., and mice were deprived water and food for 24 h. Later, water (two drinking bottles containing purified water or 2% sucrose solution, respectively, with positions switched every 12 h) and food were reintroduced. Finally, at 8:00 a.m. on the third day, the consumption of purified water and sucrose solution by each mouse was measured.

### 2.4 Behavioral Testing of Forced Swimming Test

Forced swimming test (FST) was conducted according to the previously developed protocol (Sekio and Seki 2014). A glass cylinder (30 cm in height and 10 cm in diameter) was filled with water to a depth of 25 cm at a temperature of 25 ± 1°C. Each mouse was individually placed into the cylinder, and their behavior was observed for 6 min. The time of struggling or remaining immobile was recorded. Immobility was defined as the mice remaining still or slightly moving only with one front paw to maintain equilibrium. At the end of this test, mice were immediately removed from the cylinder, dried thoroughly, and returned to their cages. The water in the cylinder was replaced for each subsequent trial to ensure cleanliness.

### 2.5 Animal Dissection

Mice were anesthetized with CO_2_. We hereby declare that all of our handling methods were performed in strict accordance with the ARRIVE guidelines (https://arriveguidelines.org). Serum was collected via the ocular vein, followed by euthanasia with neck dissection. Three mouse brain tissues were fixed with 4% paraformaldehyde via cardiac perfusion and stored at room temperature (RT), while other mouse brain tissues were directly preserved at −80°C.

### 2.6 Hematoxylin and Eosin Staining and Immunohistochemistry Detection

Mouse brain tissue sections were dehydrated, rehydrated, and used for hematoxylin and eosin (HE) staining and immunohistochemistry (IHC) analysis. For IHC, sections were incubated in a 3% H_2_O_2_ solution at RT for 15 min to quench endogenous peroxidase activity. After rinsing with distilled water, sections were subjected to antigen repair, then blocked with normal goat serum at RT for 30 min. After removing the blocking solution, sections were incubated with primary antibodies included Prdx1, Prdx2, Prdx4, and Prdx5  at 4°C overnight respectively. Sections were warmed to RT on the next day, and washed with PBS for three times, then incubated with secondary antibodies at RT for 1.5 h. Subsequently, sections were stained with DAB, counterstained with hematoxylin, dehydrated, cleared, and mounted. Finally, sections were examined under a microscope.

### 2.7 Establishment of LPS‐Induced Inflammation Model in BV2 Cells

BV2 cells, derived from immortalized mouse microglia (CL‐0493, Pricella, China), were cultured in an incubator at 37°C and 5% CO_2_. Cells were maintained in DMEM (Pricella, China) supplemented with 1% penicillin/streptomycin and 10% fetal bovine serum (FBS, Pricella, China). BV2 cells were seeded with a density of 1 × 10^6^ cells per well in a six‐well plate and cultured for 24 h. Cells were then divided into two groups: the control group and the LPS group. After washing twice with PBS, the control group was maintained in complete medium, while the LPS group was exposed to complete medium containing 1 µg/mL LPS for 24 h.

### 2.8 Enzyme‐Linked Immunosorbent Assay

The levels of transforming growth factor‐beta (TGF‐β1) (Rui Xin Biotech, No. RXW202402M, China), interleukin‐1 beta (IL‐1β) (Rui Xin Biotech, No. RXW203063M, China), and TNF‐α (Rui Xin Biotech, No. RXW202412M, China) in mice serum and BV2 cells supernatant were measured using enzyme linked immunosorbent assay (ELISA) kits. The absorbance was measured at 450 nm using a Varioskan LUX microplate reader (Darui Kangti, Guangzhou, China).

### 2.9 Detection of NO and ROS Levels in BV2 Cells

The culture medium of the control and the LPS groups was removed, then ROS staining working solution (Beyotime, China) was added. After 30 min of treatment, intracellular ROS expression was observed under a fluorescence microscope. Images from each group were collected. BV2 cells supernatant was dealt with NO detection kit (Beyotime, China), then NO levels were measured in multifunctional microplate reader.

### 2.10 Real‐Time Quantitative Polymerase Chain Reaction

Total RNA was extracted from mouse hippocampus tissues and BV2 cells using TRIzol reagent (Monad, No. M120201S, China). The RNA concentration and purity were measured in a microspectrophotometer (KRIRO, No. K5600C, China). Subsequently, RNA was reverse‐transcribed into cDNA via a reverse transcription kit (Yugong Biotech, No. EG20117M, China). Real‐time quantitative polymerase chain reaction (RT‐qPCR) was performed using 2X Q3 SYBR Green SuperMix (TOLOBIO, No. 22204‐01, China) on a Roche LightCycler 480 Real‐time PCR system (Germany). The reaction mixture consisted of cDNA, forward primer, reverse primer, ddH_2_O, and SYBR Green SuperMix, with a final reaction volume of 20 µL. The thermal cycling conditions were as follow: initial denaturation at 95°C for 30 s, followed by 40 cycles of 95°C for 5 s and 60°C for 3 s. mRNA expression was normalized to the internal reference gene GAPDH using the 2^−ΔΔCT^ method. Each experiment was independently repeated three times to ensure data reliability. The primer sequences are detailed in Table 1.

**TABLE 1:** Primers.

Gene	Primer	Sequence (5′–3′)
Prdx1	Forward	TTTACCCTCTTGACTTTACT
	Reverse	ATCCTCCTTGTTTCTTGG
Prdx2	Forward	GAGGGCATTGCTTACAGG
	Reverse	TACAGAGCGTCCCACAGG
Prdx4	Forward	TCTAAGCAAAGCCAAGAT
	Reverse	TCCCACGATAGTCAGTCA
Prdx5	Forward	GTTCAAGGGCAAGAAAGGT
	Reverse	ACGTCATTAACGCTCAGACA
GAPDH	Forward	GGTTGTCTCCTGCGACTTCA
	Reverse	TGGTCCAGGGTTTCTTACTCC

### 2.11 Western Blotting

Total protein was extracted from mouse hippocampus tissues and BV2 cells and quantified via the BCA Protein Assay Kit (Beyotime, Nanjing, China). Denatured proteins were separated by SDS‐PAGE and subsequently transferred onto a PVDF membrane. The membrane was blocked with 5% skim milk in TBST for 1 h at RT, followed by incubation with primary antibodies overnight at 4°C: GAPDH (Proteintech, No. 81640‐5‐RR, China), Prdx1 (Proteintech, No. 515816‐1‐AP, China), Prdx2 (Proteintech, No. 10545‐2‐AP, China), Prdx4 (ABonal, No. 4000001442, China). After PBST washing for three times, the membrane was incubated with the corresponding secondary antibody for 1 h at RT. Protein expression levels were analyzed by measuring the band intensity using ImageJ Software (Version 1.54).

### 2.12 Data Analysis

Statistical analysis was performed using GraphPad Prism 10.2.0. Measurement data that followed a normal distribution were presented as mean ± standard deviation (Mean ± SD). To compare results between two groups in western blotting (WB), RT‐qPCR, OFT, FST, and SFT, we used the Student *t*‐test. A *p*‐value < 0.05 was considered statistically significant.

## 3 Results

### 3.1 Behavior Analysis

**FIGURE 1:**
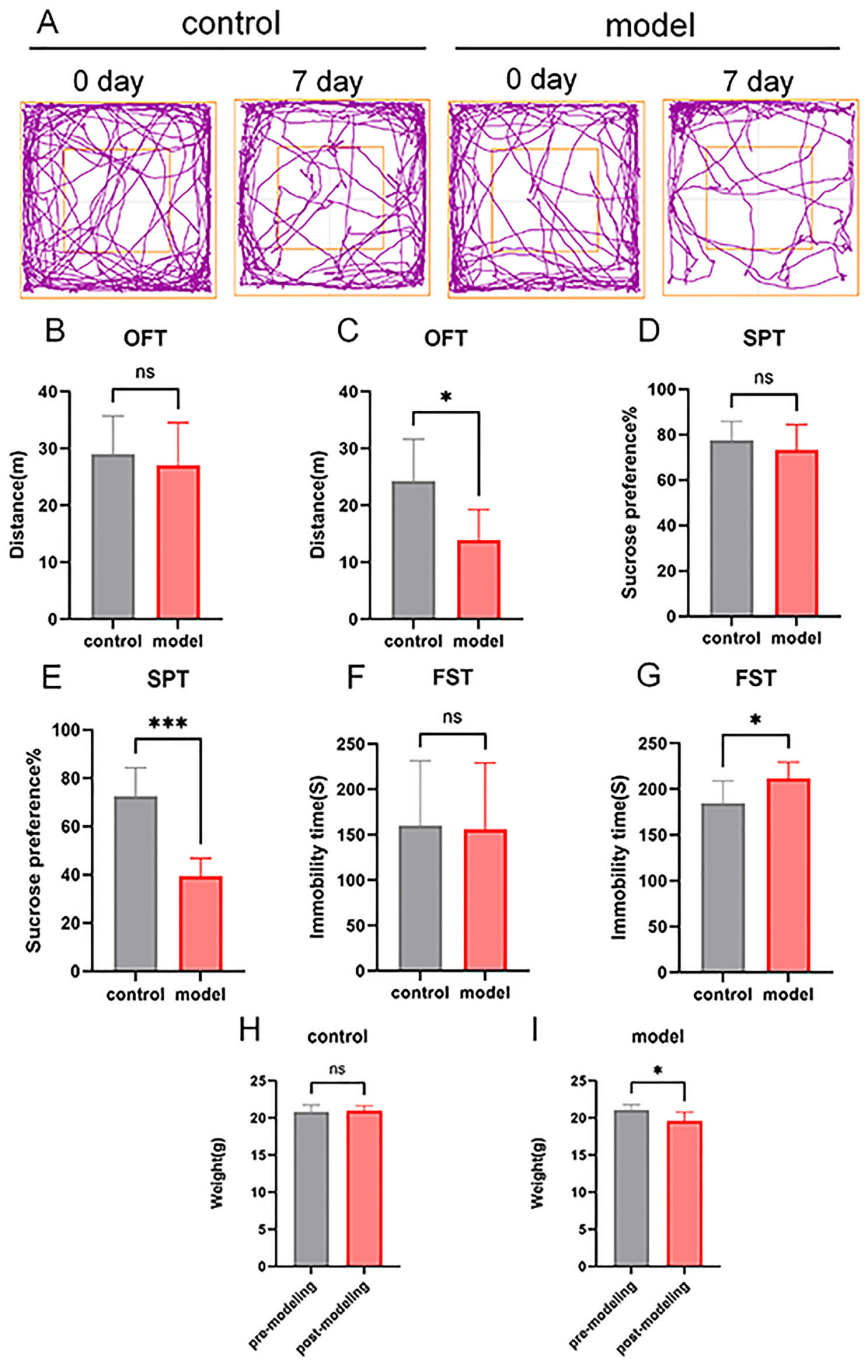
Comparative analysis of animal behavioral patterns pre‐ and post‐modeling. (A) Trajectory of the open field test (OFT); (B) total duration in the OFT before modeling (control vs. LPS group); (C) total duration in the OFT after modeling (control vs. LPS group); (D) sucrose preference test (SPT) rate before modeling (control vs. LPS group); (E) SPT rate after modeling (control vs. LPS group); (F) immobility time in the forced swimming test (FST) before modeling (control vs. LPS group); (G) immobility time in the FST after modeling (control vs. LPS group); (H) body weight difference before modeling (control vs. LPS group); (I) body weight difference after modeling (control vs. LPS group). “ns” suggests no statistical difference, *p *> 0.05; *p < 0.05; ***p < 0.001.

The sucrose preference test (SPT), FST, OFT, and body weight measurements were conducted to evaluate LPS‐induced depression in mice. Figure 1A illustrates the movement trajectories of mice on Day 0 and Day 7. Compared with the control group, no significant differences were observed in the LPS group in OFT total duration (Figure 1B), sucrose preference rate in SPT (Figure 1D), FST immobility time (Figure 1F), and body weight (Figure 1H) on Day 0 (all *p *> 0.05).

On Day 7, compared with the control group, the LPS group exhibited a significant decrease in OFT total duration (*p *< 0.05, Figure 1C). In addition, in the LPS group, the sucrose preference rate in SPT was significantly reduced (*p *< 0.05, Figure 1E), and the immobility time in FST was significantly prolonged when compared with the control group (*p *< 0.05, Figure 1G). Furthermore, the body weight of the LPS group was significantly lower than the control group (*p *< 0.05, Figure 1I). These findings indicated that LPS‐induced mice displayed behavioral characteristics consistent with depression‐like symptoms.

### 3.2 Pathological Changes in Neurons and Neuroinflammation Levels in LPS‐Induced Depression Mouse Model

**FIGURE 2:**
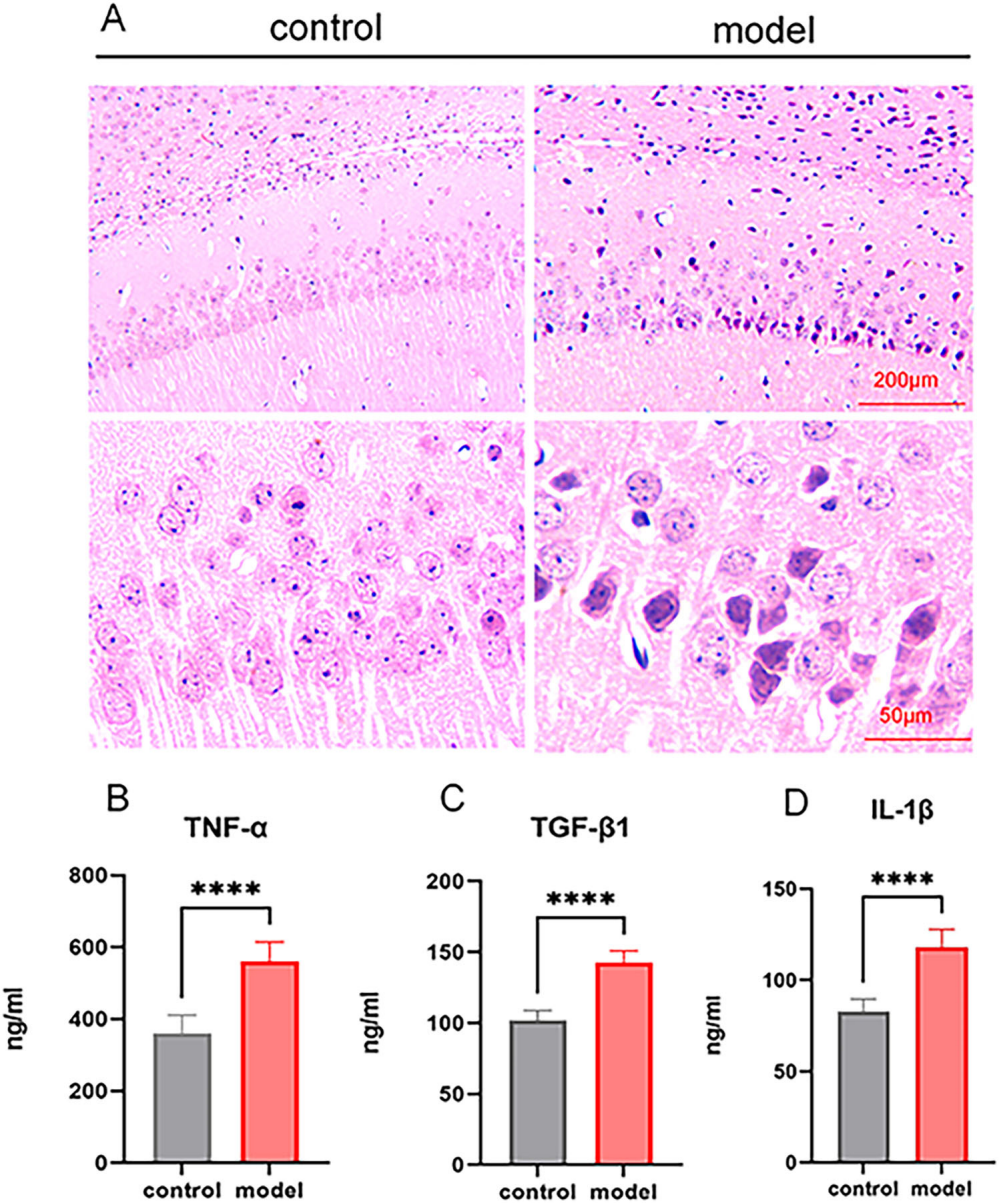
Pathological changes and serum inflammatory factor levels in LPS‐induced ICR mouse depression model. (A) HE staining of mice brain tissues; (B) tumor necrosis factor‐alpha (TNF‐α) levels in mice serum; (C) transforming growth factor‐beta1 (TGF‐β1) levels in mice serum; (D) interleukin‐1 beta (IL‐1β) levels in mice serum; ****p < 0.0001.

HE staining results revealed that the control group exhibited normal brain morphological character with round nuclei, large cell bodies, and well‐organized neurons. In contrast, LPS‐induced mice showed significant histological alterations, including cell body atrophy, nuclear condensation, cellular swelling, interstitial edema, and disorganized neuronal arrangement (Figure 2A).

ELISA was employed to measure mouse serum cytokine levels, including TGF‐β1, IL‐1β, and TNF‐α. The results demonstrated that, compared with the control group, the LPS group exhibited significant elevation in serum TNF‐α (Figure 2B, ****p < 0.0001), TGF‐β1 (Figure 2C, ****p < 0.0001), and IL‐1β (Figure 2D, ****p < 0.0001). These findings suggested a marked increase in neuroinflammatory levels in LPS‐induced mice.

### 3.3 Expression of Prdx1, Prdx2, Prdx4, and Prdx5 in the Hippocampus CA1 Region of LPS‐Induced Depression Mouse Model

**FIGURE 3:**
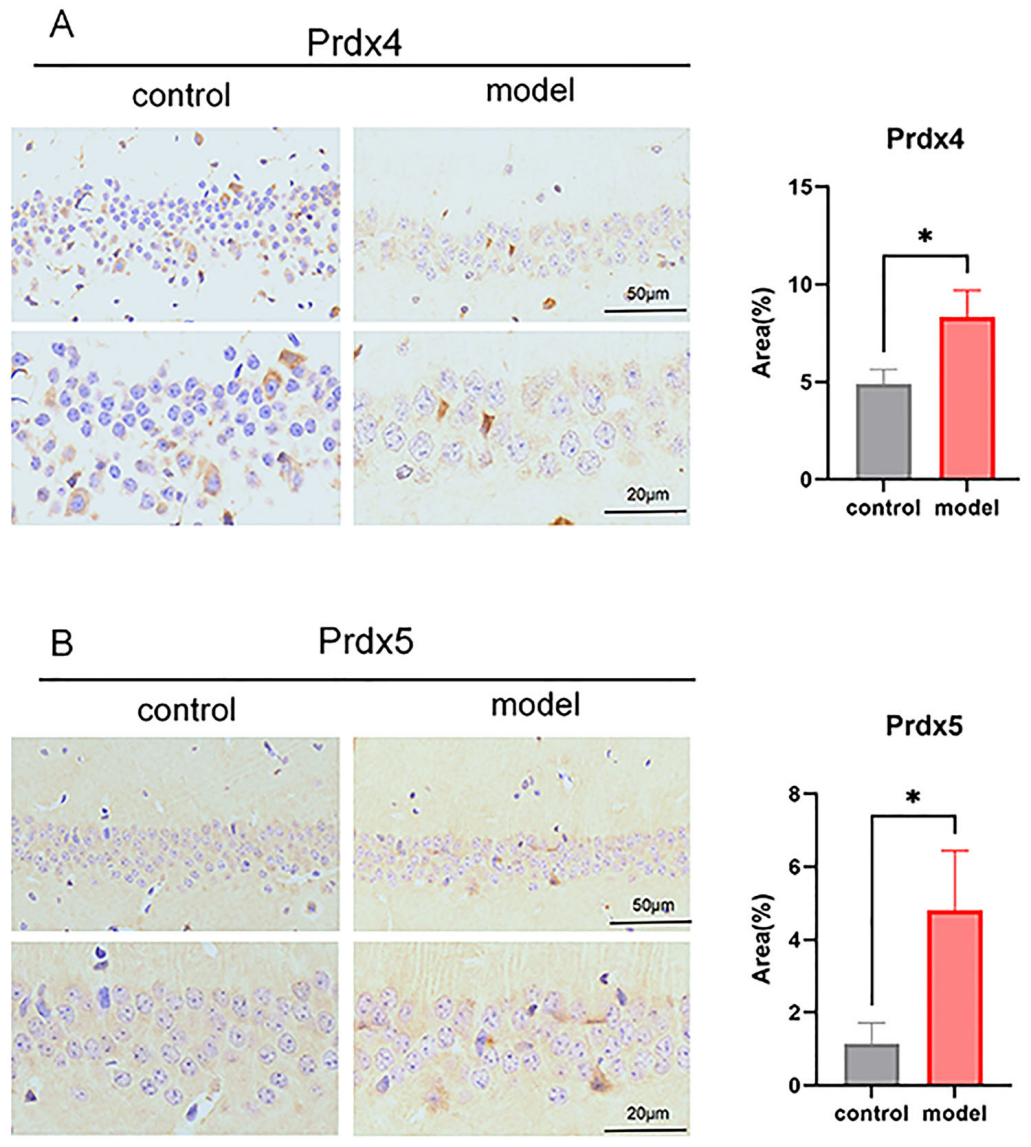
Immunohistochemistry detected the protein expression of Peroxiredoxin1 (Prdx1) and Prdx2 in brain tissues. (A) Prdx1 immunohistochemistry staining and expression levels in brain tissues; (B) Prdx2 immunohistochemistry staining and expression levels in brain tissues; ns, no significant difference, *p *> 0.05.

**FIGURE 4:**
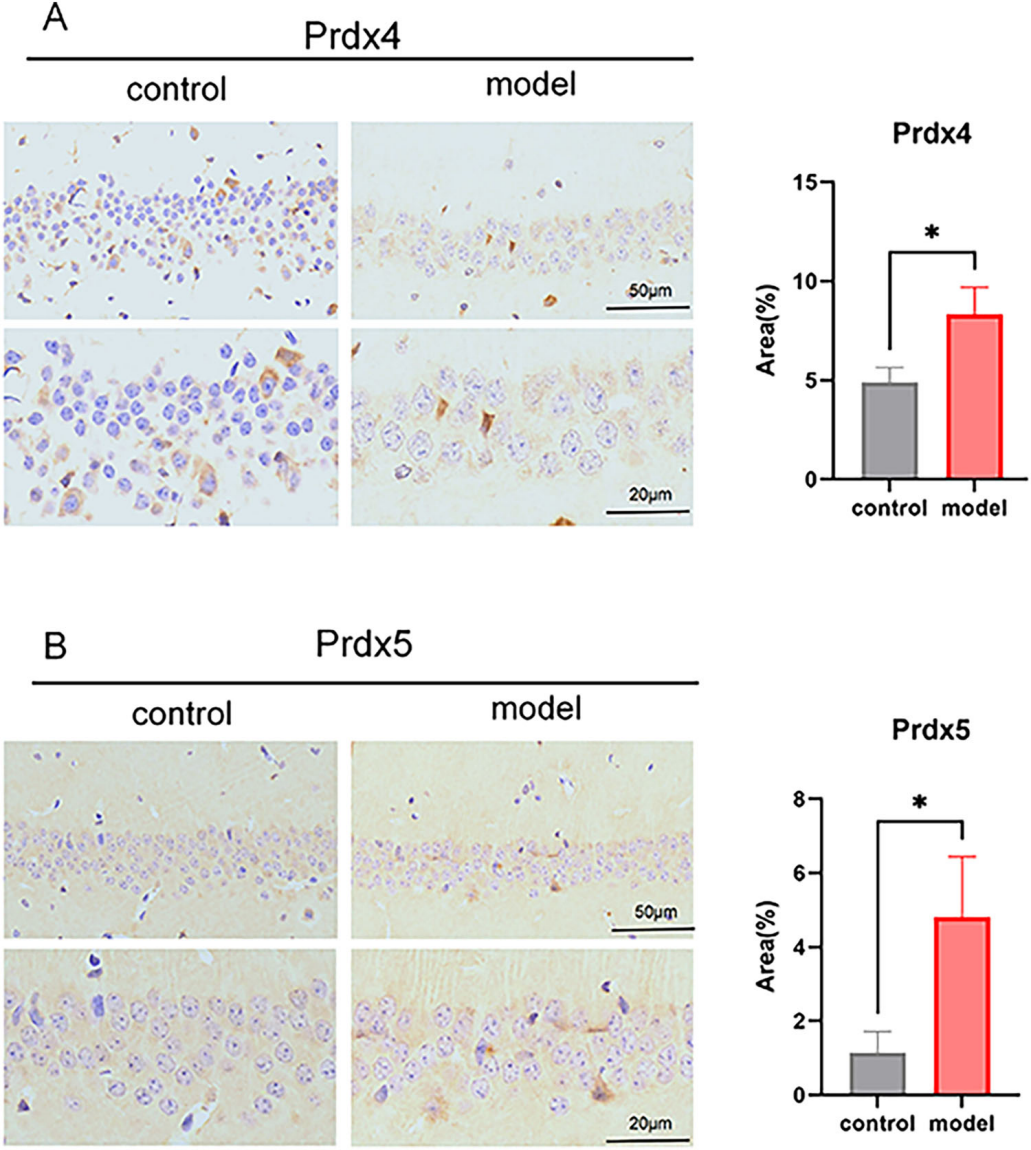
Immunohistochemistry detected the protein expression of Peroxiredoxin4 (Prdx4) and Prdx5 in brain tissues. (A) Prdx4 immunohistochemistry staining and expression levels in brain tissues; (B) Prdx5 immunohistochemistry staining and expression levels in brain tissues; ns suggests no statistical difference, *p *> 0.05;*p < 0.05.

**FIGURE 5:**
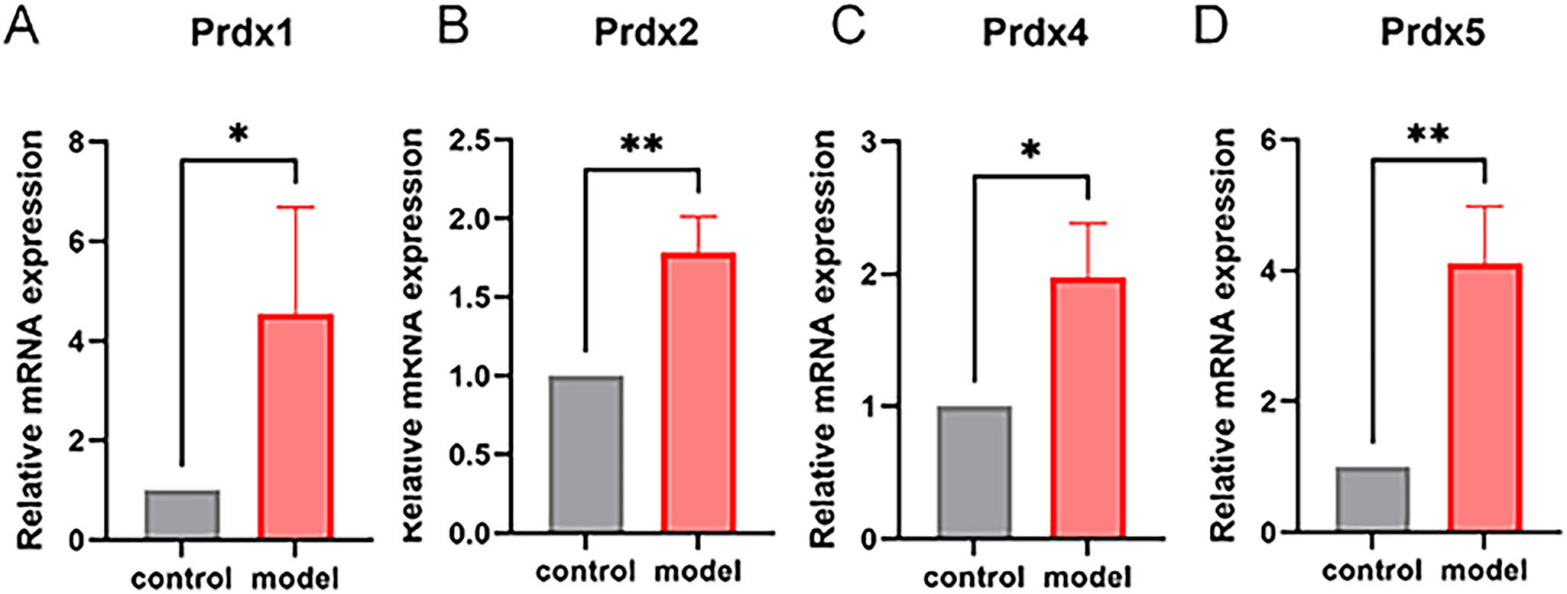
RT‐qPCR detected the mRNA expression of Peroxiredoxin1 (Prdx1), Prdx2, Prdx4, and Prdx5 in brain tissues. (A) mRNA expression levels of *Prdx1* in brain tissues; (B) mRNA expression levels of *Prdx2* in brain tissues; (C) mRNA expression levels of *Prdx4* in brain tissues; (D) mRNA expression levels of *Prdx5* in brain tissues; *p < 0.05; **p < 0.01.

**FIGURE 6:**
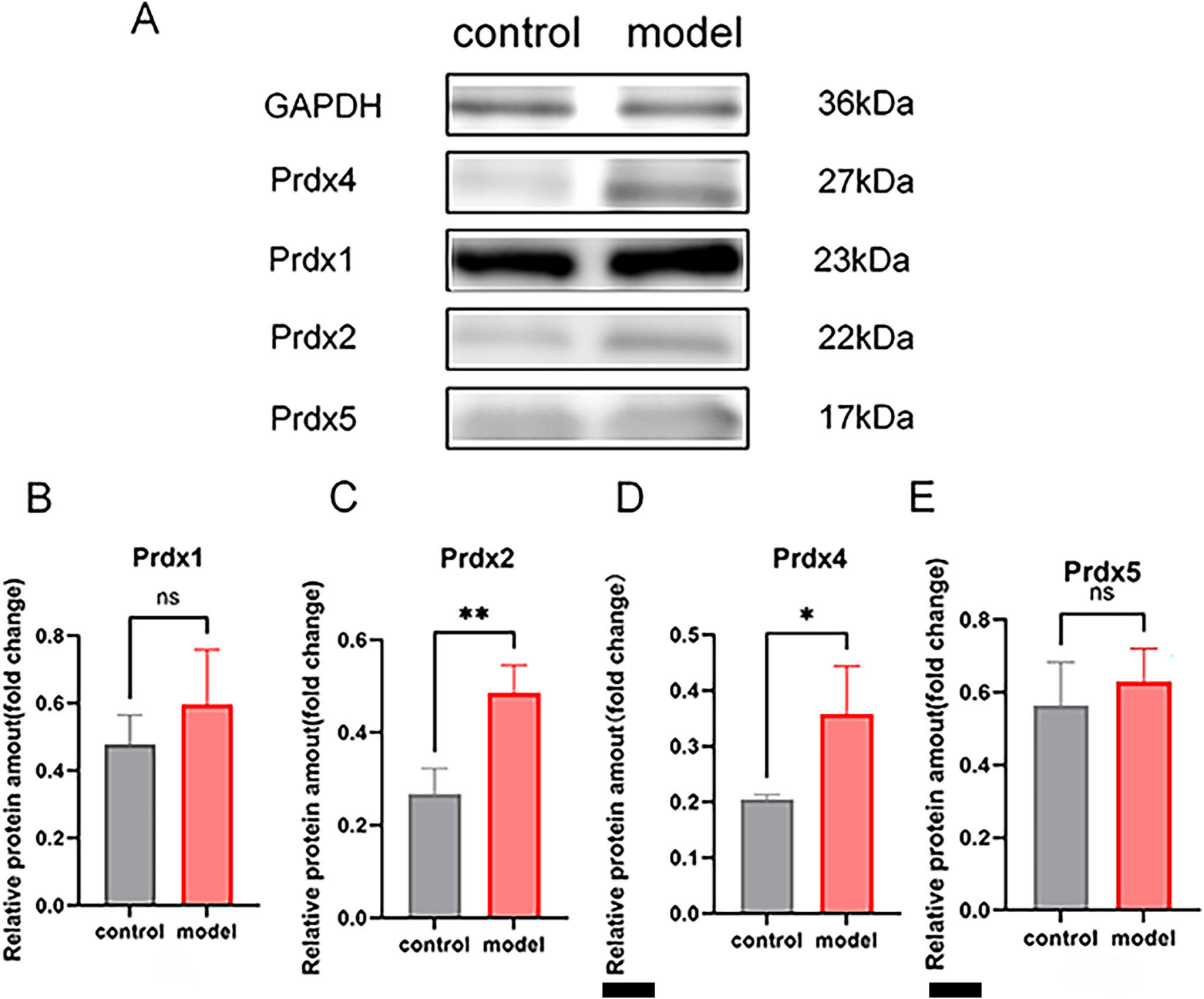
WB detected the protein expression of Peroxiredoxin1 (Prdx1), Prdx2, Prdx4, and Prdx5 in brain tissues. (A) Protein expression bands; (B) Prdx1 protein expression levels; (C) Prdx2 protein expression levels; (D) Prdx4 protein expression levels; (E) Prdx5 protein expression levels; ns, no significant difference, *p *> 0.05; *p < 0.05; **p < 0.01.

IHC results suggested that, when compared with the control group, the LPS group showed no significant differences in Prdx1 (Figure 3A) and Prdx2 (Figure 3B). However, there was a significant increase in Prdx4 and Prdx5 expression in the LPS group (Figure 4, *p < 0.05). RT‐qPCR results are shown in Figure 5. Compared with the control group, mRNA expression levels of Prdx1 (Figure 5A), Prdx2 (Figure 5B), Prdx4 (Figure 5C), and Prdx5 (Figure 5D) were significantly increased in the LPS group (*p < 0.05, **p < 0.01). WB was used to detect protein expression of Prdx1, Prdx2, Prdx4, and Prdx5. Compared with the control group, the protein expression levels of Prdx2 (Figure 6C) and Prdx4 (Figure 6D) were significantly increased in the LPS group (*p < 0.05), but Prdx1 (Figure 6B) and Prdx5 (Figure 6E) were elevated in the LPS group with no statistical significance (Figure 6E, ^ns^ *p *> 0.05).

### 3.4 Inflammatory Factors and Oxidative Stress in LPS‐Induced BV2 Cells

**FIGURE 7:**
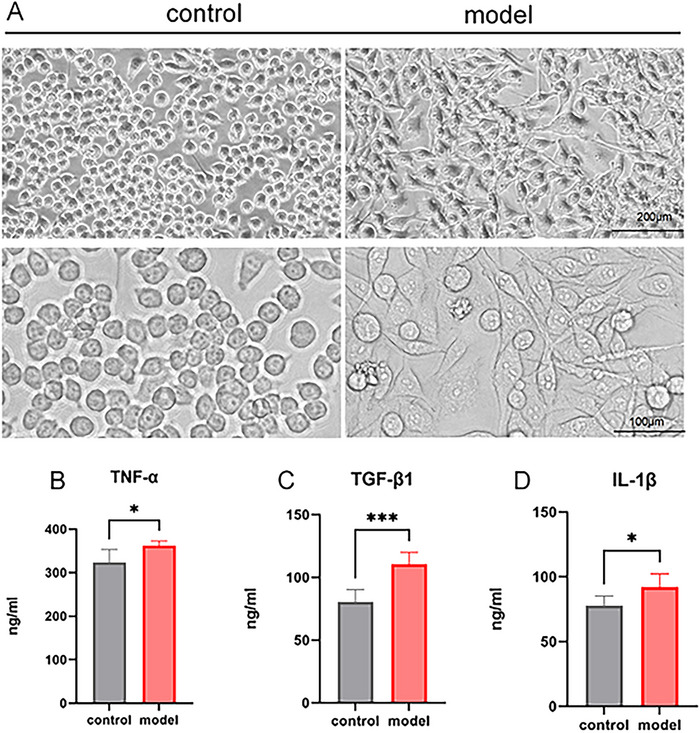
LPS‐induced morphological changes and inflammatory levels in BV2 cells. (A) Morphology of BV2 cells; (B) tumor necrosis factor‐alpha (TNF‐α) levels in cell supernatant; (C) transforming growth factor‐beta1 (TGF‐β1) levels in cell supernatant; (D) interleukin‐1 beta (IL‐1β) levels in cell supernatant. *p < 0.05, ***p < 0.001.

In the control group, BV2 cells exhibited typical morphology characterized by rounded and relatively small cell bodies, minimal intracellular vacuoles, and well‐defined edges. In contrast, LPS‐induced BV2 cells in an activated state displayed enlarged cell bodies with “amoeboid” shape, cellular thickening, numerous intracellular vacuoles, and less compact membranes (Figure 7A). ELISA results demonstrated that the LPS group had markedly higher TNF‐α (Figure 7B, *p < 0.05), TGF‐β1 (Figure 7C, ***p < 0.001), and IL‐1β (Figure 7D, *p < 0.05) levels than the control group.

**FIGURE 8:**
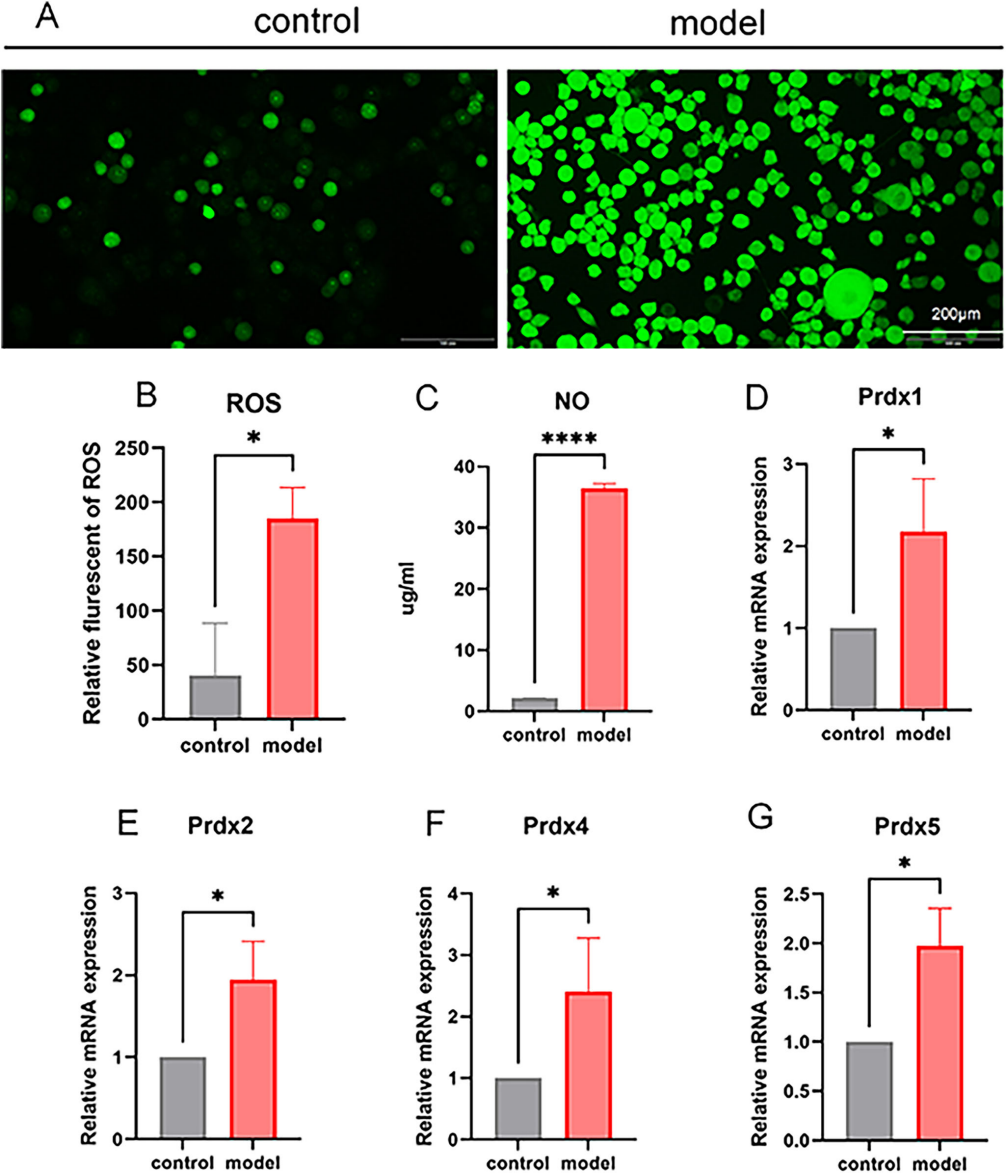
The expression levels of Prdxs, reactive oxygen species (ROS), and nitric oxide (NO) in BV2 cells. (A) ROS fluorescence images; (B) ROS fluorescence levels in BV2 cells; (C) NO levels in BV2 cell supernatant; mRNA expression levels of *Prdx1* (D), *Prdx2* (E), *Prdx4* (F), and *Prdx5* (G) in BV2 cells; ns, no significant difference, *p *> 0.05; *p < 0.05; ****p < 0.0001.

The LPS group exhibited a significant increase in ROS expression compared with the control group (Figure 8A,B, *p < 0.05). The NO detection was used to measure the inflammatory factor level in the cell supernatant. Compared with the control group, the LPS group showed significantly elevated NO expression (Figure 8C, ****p < 0.0001). These findings suggested the occurrence of neuroinflammation in LPS‐induced BV2 cells.

### 3.5 Expression of Prdx1, Prdx2, Prdx4, and Prdx5 in LPS‐Induced BV2 Cells

RT‐qPCR was employed to measure mRNA expression levels of Prdx1, Prdx2, Prdx4, and Prdx5 in BV2 cells. Compared with the control group, the LPS group showed significantly higher mRNA expression levels of Prdx1 (Figure 8D), Prdx2 (Figure 8E), Prdx4 (Figure 8F), and Prdx5 (Figure 8G) (*p < 0.05, ****p < 0.0001).

**FIGURE 9:**
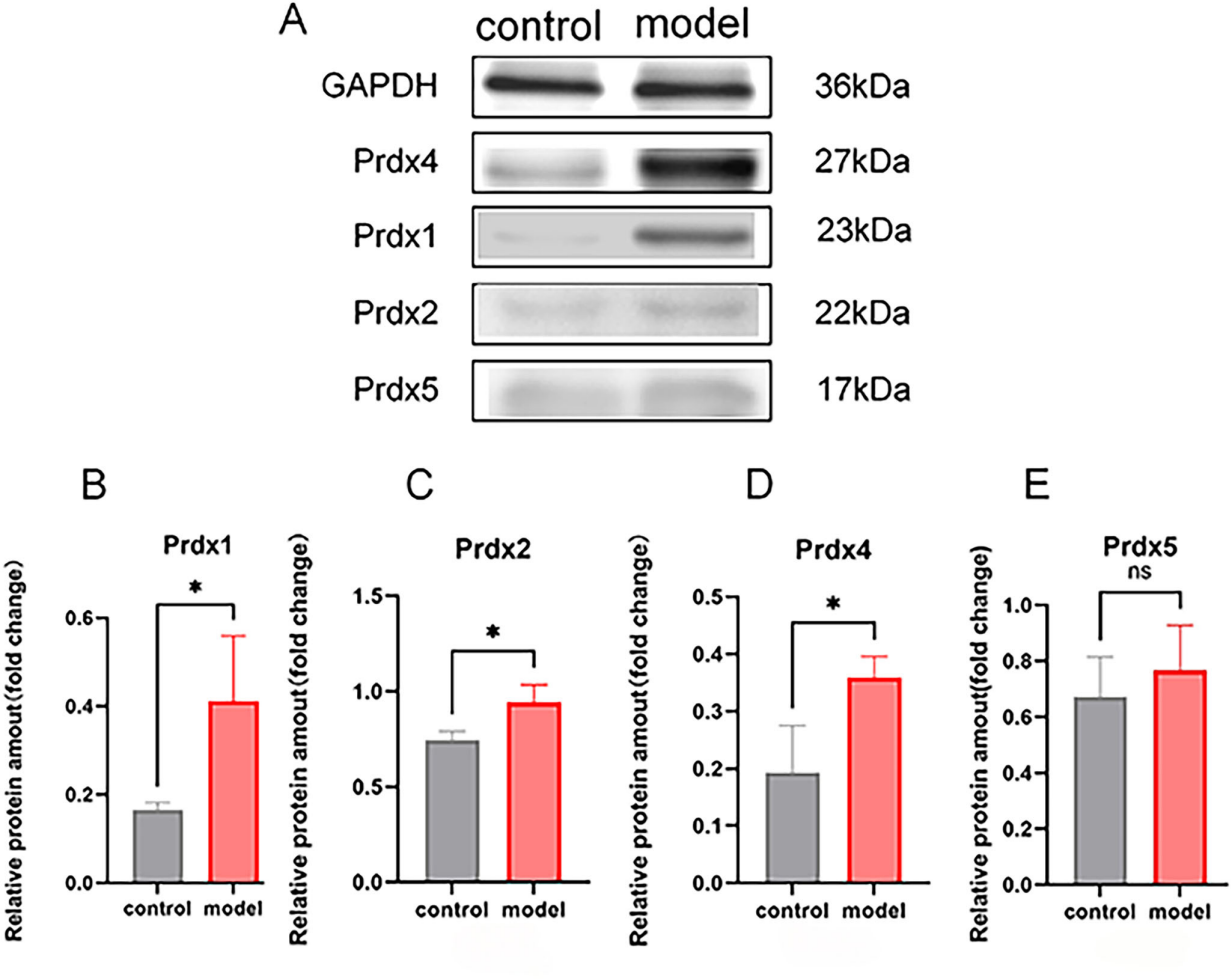
WB detected the protein expression of Peroxiredoxin1 (Prdx1), Prdx2, Prdx4, and Prdx5 in BV2 cells. (A) Protein expression bands; protein expression levels of Prdx1 (B), Prdx2 (C), Prdx4 (D), and Prdx5 (E) in BV2 cells; ns, no significant difference, *p *> 0.05; *p < 0.05.

WB results (Figure 9) revealed that when compared the control group, the protein expression levels of Prdx1, Prdx2, and Prdx4 in the LPS group were significantly increased (Figure 9B–D, *p < 0.05), and the protein expression level of Prdx5 was elevated in LPS‐induced BV2 cells with no significant difference (Figure 9E, ^ns^ *p *> 0.05).

## 4 Discussion

Depression is a mental disorder characterized by spontaneous and persistent low mood, with high rates of disability, mortality, and recurrence, severely impacting social functioning (O'Rourke et al. 2023). Inflammation plays a crucial role in the occurrence and progression of depression through regulating immune factor production, immune cell activation, neurogenesis, synaptic plasticity, and neurotransmission (Zheng et al. 2025; Wu and Zhang 2023). ROS is essential in cellular signaling and defense against invading microorganisms. Excessive ROS generation, coupled with the depletion of antioxidant defenses, disrupts redox homeostasis, leading to pro‐inflammatory signaling activation, which ultimately results in cellular necrosis (Shen et al. 2024). Bhatt et al. (2020) have demonstrated that oxidative stress is a key triggering factor for MDD. Altered redox balance and ROS accumulation have been consistently observed in patients with depression and anxiety disorders, as well as in depression animal models (K. Li et al. 2020). Prdxs have been reported to participate in posttranslational modifications, balancing ROS signaling and stress responses (T. Qiu et al. 2025). However, the relationship among Prdxs, ROS, and depression remains to be elucidated.

In this study, we investigated the roles and mechanisms of Prdx1, Prdx2, Prdx4, and Prdx5 in depression based on LPS‐induced mouse depression model and BV2 cells neuroinflammation model. After modeling, LPS group mice exhibited a significant decrease in total duration (OFT), sucrose preference rate (SPT), and body weight, but an increase in immobility time (FST) than the control group. These results indicated that LPS‐induced mice displayed behavioral characteristics consistent with depression‐like symptoms. In addition, inflammatory factors, such as IL‐1β and TNF‐α, were significantly increased in the serum of LPS‐induced mice. C. P. Liu et al. (2021) found that the expression levels of ionized calcium binding adapter molecule 1 (Iba‐1), inducible NO, IL‐1β, and TNF‐α were significantly increased in depressed mice.

Animal studies conducted by Guo et al. (2023) have shown that chronic unpredictable mild stress (CUMS) could induce pro‐inflammatory cytokines in the medial prefrontal cortex (mPFC), with increased TNF‐α positive microglia in the prefrontal region of stress‐susceptible mice. Zheng et al. further demonstrated that microglial activation and elevation of pro‐inflammatory cytokines in the lateral amygdala, coupled with enhanced presynaptic glutamate release, resulted in an excitatory/inhibitory (E/I) imbalance, leading to anxiety‐ and depression‐like behaviors in LPS‐induced neuroinflammatory mouse (Zheng et al. 2021). Clinical studies by Pan et al. also revealed that pro‐inflammatory markers such as IL‐1β, interleukin‐6 (IL‐6), TNF‐α, and C‐reactive protein (CRP) were significantly upregulated in elderly patients with depression and Alzheimer's disease (Pan et al. 2014). Our results were consistent with these findings. Lama et al. (2022) further demonstrated that both depressed patients and animals typically exhibited elevated levels of inflammatory cytokines, including IL‐1β, IL‐6, and TNF‐α.

RT‐qPCR and WB results suggested the mRNA and protein expression levels of Prdx1, Prdx2, Prdx4, and Prdx5 in brain tissues were significantly elevated. IHC analysis further confirmed that Prdx1 in hippocampus cornu ammonis 1 (CA1) region of mice increased without a significant difference, and Prdx4 as well as Prdx5 increased significantly, but Prdx2 decreased without statistical significance. These might suggest potential posttranscriptional regulation. Prdx1 and Prdx5 are classified as stress‐inducible proteins, and their transcription is jointly regulated by E twenty‑six(Ets) family transcription factors and high mobility group box 1 (HMGB1) protein (Shiota et al. 2008). The mRNA levels could be easily affected by the cell stress state and changes rapidly, but the accumulation of protein levels might take time or be restricted by more factors. A study (Shan et al. 2005) on the development of mouse embryonic limbs found that the expression of Prdx1 was downregulated during programmed cell death, and its mRNA and protein levels were not completely synchronized, suggesting the existence of posttranscriptional regulation.

To further investigate the roles of Prdx1, Prdx2, Prdx4, and Prdx5 in the development of neuroinflammation and depression, we established an LPS‐induced neuroinflammatory BV2 microglial cell model. Results demonstrated that the levels of NO, IL‐1β, and TNF‐α in the cell supernatant of the LPS group were significantly elevated, indicating the occurrence of inflammation. Concurrently, the mRNA and protein levels of Prdx1, Prdx2, Prdx4, and Prdx5 in LPS group cells also increased significantly. Furthermore, we found that the ROS levels in the LPS group were markedly higher than the control group. Therefore, during the development of depression, elevated neuroinflammation and ROS levels were accompanied by the increased expression levels of Prdx1, Prdx2, Prdx4, and Prdx5, indicating a positive correlation between Prdxs and the occurrence of depression.

Studies have reported that Prdx1 was positively correlated with both DASS‐21 depression scores and stress scores (Y. Zhang et al. 2021). As a damage‐associated molecular pattern (DAMP), Prdx2 accelerated post‐stroke brain injury by activating inflammatory responses (J. Liu et al. 2020). Z. Wang et al. (2025) found the upregulation of antioxidant genes (Prdx5, etc.) in mouse colons following DSS treatment, suggesting excessive oxidative stress during intestinal inflammation. Interestingly, Prdx4 mitigated microvascular inflammation and reduced the infiltration of destructive neutrophils and pro‐inflammatory macrophages into the brain parenchyma after I/R. These demonstrated that Prdx4 was indispensable in protecting the blood–brain barrier (BBB) and promoting functional recovery following I/R‐induced brain injury (Yang et al. 2021).

However, studies also reported that Prdx2 could protect neurons from oxidative stress and inflammatory damage by catalyzing hydrogen peroxide (H_2_O_2_) to reduce ROS production and participating in the regulation of various neuronal signaling pathways (J. Liu et al. 2020). In addition, Park et al. (2016) found that Prdx5 alleviated neuroinflammation by modulating LPS‐induced mitochondrial fission through the clearance of NO*_x_*‐derived and cytosolic ROS, thereby regulating Ca^2+^‐calcineurin‐dependent Drp1 dephosphorylation. Therefore, elevation of inflammatory factors and ROS in BV2 cells, accompanied by upregulation of Prdx1, Prdx2, Prdx4, and Prdx5, might indicate cellular oxidative stress and inflammatory injury. Prdxs could eliminate excessive ROS and alleviate the inflammatory response caused by oxidative stress during depression.

Furthermore, results showed that the upregulation of Prdx1, Prdx2, Prdx4, and Prdx5 was accompanied with elevated levels of TGF‐β1 in the LPS‐induced mice serum and BV2 cells supernatant. TGF‐β1, a member of the TGF family, is involved in the development of stress‐ and depression‐related mechanisms and plays a critical role in modulating inflammatory responses in depression as well as restoring imbalances of various cytokines (Y. Li et al. 2023). TGF‐β1 could be used as a predictive biomarker for adult depression resulting from early‐life trauma, such as childhood abuse and life stressors (A. Qiu et al. 2021; Cattaneo et al. 2018). Studies by Davami et al. (2016) and Momeni et al. (2014) revealed that peripheral TGF‐β/TGF‐β1 levels in patients with depression were significantly higher than those in healthy controls.

In summary, this study revealed that Prdx1, Prdx2, Prdx4, and Prdx5 were elevated in depression models, which might relate to the occurrence of neuroinflammation, coupled with upregulation of oxidative stress responses. The upregulation of Prdxs is plausibly a protective/compensatory response to LPS‐induced oxidative stress. However, the mechanisms by which Prdxs participated in neuroinflammation and oxidative stress remain to be further investigated. The sample size (*n* = 6, per group) in this study might decrease the statistical power. Further experiments with sufficient statistical effect should be carried out to reduce this limitation and explore the isoform‐specific and cross‐brain‐region distribution differences of Prdx family members in neuroinflammation, offering new therapeutic targets for depression.

## 5 Conclusion

Depression is a chronic psychiatric disorder that severely impacts social functioning, with inflammation playing a significant role in its onset and progression. We successfully established the ICR depression mouse model and the BV2 cell inflammatory model. This study revealed that LPS upregulated the levels of TGF‐β1, IL‐1β, and TNF‐α and elevated the intracellular ROS, NO, and Prdxs expression. These suggested that the upregulation of Prdxs might be related to increased inflammatory and oxidative stress responses during depression. This study provided new strategies for the treatment of depression.

## Author Contributions

**Zhifang Zhang**: data curation, investigation, methodology. **Nanshi Li**: conceptualization, supervision, writing – review and editing, project administration, funding acquisition. **Mingkun Liang**: writing – review and editing, supervision. **Fangyan Qin**: writing – review and editing, supervision. **Xueli Shi**: supervision, methodology. **Kaihua Wang**: writing – review and editing, supervision. **Qijing Qin**: methodology. **Qing He**: writing – review and editing. **Ying Jiang**: methodology, project administration, writing – review and editing, writing – original draft, data curation. **Hui Qin**: formal analysis, writing – original draft, writing – review and editing, project administration, funding acquisition.

## Funding

This research was funded by the Guangxi Natural Science Foundation Project (No. 2025GXNSFAA069385), Guangxi Universities Young and Middle‐aged Teachers Basic Research Ability Improvement Project (No. 2020KY07010), Guangxi University of Chinese Medicine Doctoral Research Start‐up Fund Project (No. XP020090), Key Project of Guangxi Natural Science Foundation (No. 2023GXNSFDA026048), Scientific Research Start‐up Fund for Introduced Talents of Guangxi International Zhuang Medicine Hospital (No. GZ2023RC005), and National Traditional Chinese Advantageous Specialties Construction Unit—Zhuang Medical Brain Disease Department (2024; No. 90).

## Ethics Statement

All experimental protocols were approved by the Animal Studies EthicsCommittee of Guangxi University of Chinese Medicine (approval number, DW20240319‐068).

## Conflicts of Interest

The authors declare no conflicts of interest.

## Data Availability

The data used to support the findings of this study are available from the corresponding author upon request.
